# Nrf2 Negatively Regulates Melanogenesis by Modulating PI3K/Akt Signaling

**DOI:** 10.1371/journal.pone.0096035

**Published:** 2014-04-24

**Authors:** Jung-Min Shin, Mi Yoon Kim, Kyung-Cheol Sohn, So-Young Jung, Hae-Eul Lee, Jae Woo Lim, Sooil Kim, Young-Ho Lee, Myung Im, Young-Joon Seo, Chang Deok Kim, Jeung-Hoon Lee, Young Lee, Tae-Jin Yoon

**Affiliations:** 1 Department of Dermatology and Research Institute for Medical Sciences, School of Medicine, Chungnam National University, Daejeon, Korea; 2 Department of Anatomy, School of Medicine, Chungnam National University, Daejeon, Korea; 3 Department of Dermatology and Institute of Health Sciences, School of Medicine, Gyeongsang National University, Jinju, Korea; University of Texas Health Science Center at Houston, United States of America

## Abstract

Nrf2 plays a role in protection of cells against oxidative stress and xenobiotic damage by regulating cytoprotective genes. In this study, we investigated the effect of Nrf2 on melanogenesis in normal human melanocytes (NHMCs). When NHMCs were transduced with a recombinant adenovirus expressing Nrf2, melanin synthesis was significantly decreased. Consistent with this result, overexpression of Nrf2 decreased the expression of tyrosinase and tyrosinase-related protein 1. The inhibitory effect of Nrf2 was reversed by overexpression of Keap1, an intracellular regulator of Nrf2. Interestingly, Nrf2 overexpression resulted in marked activation of PI3K/Akt signaling. Conversely, inhibition of PI3K activity by treatment with wortmannin reversed the depigmentary effects of Nrf2. Taken together, these results strongly suggest that Nrf2 negatively regulates melanogenesis by modulating the PI3K/Akt signaling pathway.

## Introduction

Skin is continuously exposed to environmental insults, including toxic chemicals and ultraviolet (UV) radiation, which can induce the formation of reactive oxygen species (ROS). Since excessive oxidative stress can evoke many biological responses, such as cell senescence, severe cell damage and even neoplastic transformation, cells must develop protective mechanisms. One important defense system includes phase II detoxification enzymes, which are regulated by the transcription factor nuclear factor E2-related factor 2 (Nrf2) [1], [2]. Under non-stressful conditions, Nrf2 is sequestered with Kelch-like ECH-associated protein 1 (Keap1) in the cytoplasm, and is constantly degraded by the ubiquitin-proteasome pathway [3], [4]. When cells are exposed to oxidative stress, Nrf2 dissociates from Keap1 and translocates to the nucleus. Nrf2 heterodimerizes with small Maf proteins and binds to the antioxidant/electrophile response elements (AREs/EpREs) of phase II detoxification enzyme genes, including NADPH: quinone oxidoreductase (NQO1), glutathione S-transferase (GST), and heme oxygenase 1 (HO-1) [5]–[7]. The importance of Nrf2 in maintaining skin homeostasis is well recognized. For example, the loss of Nrf2-mediated gene expression in K14-dnNrf2 transgenic mice results in striking enhancement of chemically induced skin papillomas [8]. Additionally, ultraviolet B (UVB) irradiation leads to accelerated photoaging in Nrf2 gene-deficient mice [9].

With regard to skin pigmentation, there is limited evidence linking Nrf2 to vitiligo. Vitiligo is an acquired pigmentary disorder of the skin and mucous membranes that is characterized by loss of pigmentation. For instance, Nrf2 promoter polymorphisms are associated with an increased risk of vitiligo [10], and transcriptional upregulation of Nrf2 and downstream detoxification genes is observed in the lesional epidermal skin of subjects with vitiligo vulgaris [11]. However, the functional role of Nrf2 in melanocyte pigmentation has not yet been elucidated. In this study, we investigated the putative role of Nrf2 in cultured normal human epidermal melanocytes (NHEMs) using an adenoviral gene delivery system. We demonstrate that Nrf2 negatively regulates melanogenesis by modulating the PI3K/Akt signaling pathway.

## Results

### Effect of Nrf2 on pigmentation of NHEMs

**Figure 1:**
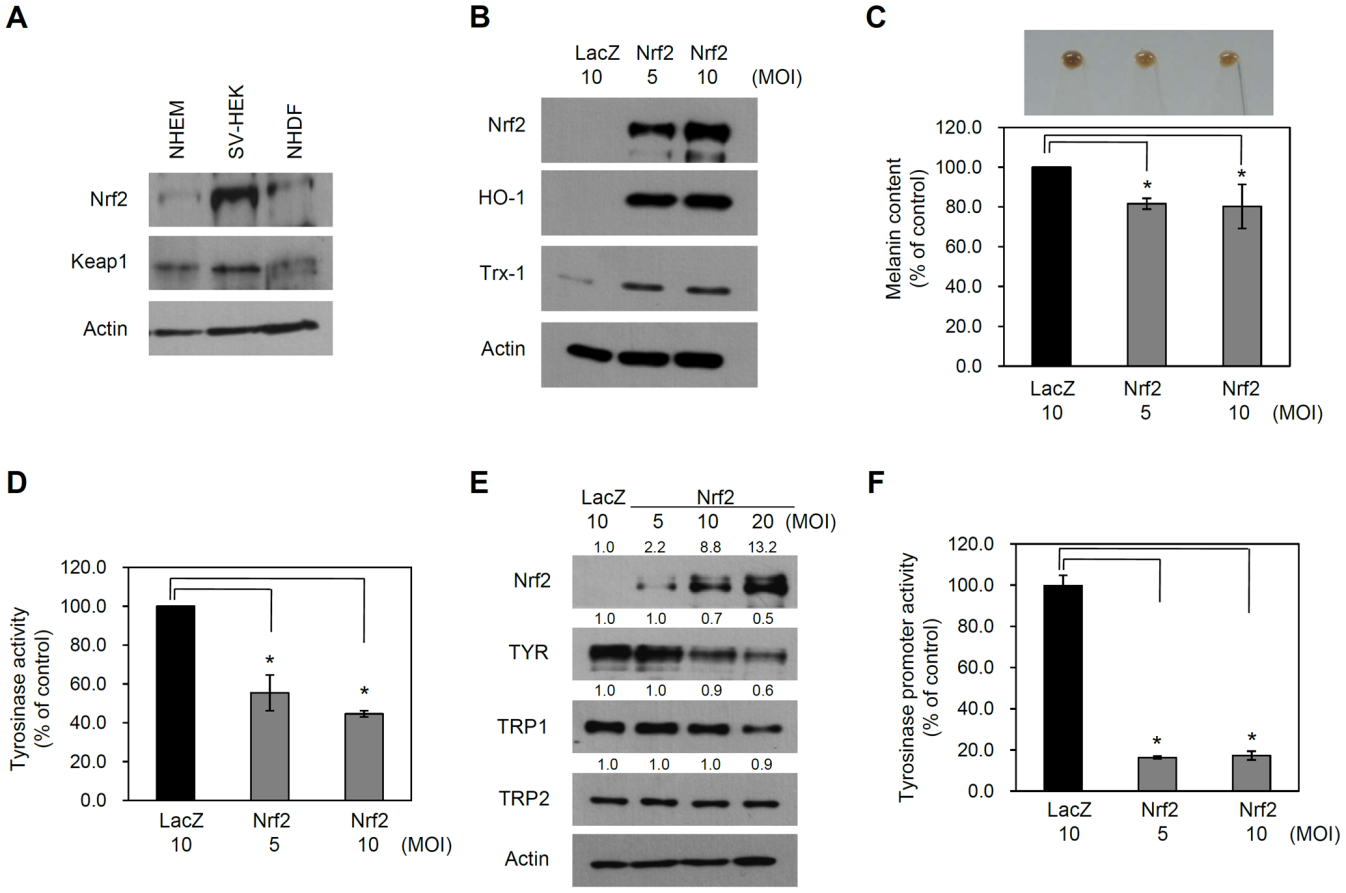
Effect of Nrf2 on melanogenesis. (A) Cellular extracts prepared from cultured normal human epidermal melanocytes (NHEMs), SV40T-transformed human epidermal keratinocytes (SV-HEK) and normal human dermal fibroblasts (NHDFs) were assessed by Western blotting. (B) NHEMs were transduced with an adenovirus expressing Nrf2 or LacZ (control) at the indicated multiplicity of infection (MOI) for 6 h. Cells were replenished and then cultured for a further 3 days. Expression of Nrf2 downstream genes was determined by Western blot analysis. (C) After adenoviral transduction, cells were harvested and spun down. Upper panel shows the pellet color. Lower panel shows melanin content measured by spectrometer. Data are the means ± SD of triplicate measurements (*P<0.01 vs. control). (D) Tyrosinase (TYR) activity was determined and expressed as a percentage of the control. Data are the means ± SD of triplicate measurements (*P<0.01 vs. control). (E) Expression levels of the melanogenic enzymes TYR, tyrosinase-related protein-1 (TRP1) and tyrosinase-related protein-2 (TPR2) were assessed by Western blotting. Actin was used as the loading control. (F) A tyrosinase promoter-luciferase reporter adenovirus was co-transduced with an adenovirus expressing Nrf2 or LacZ (control). The cells were harvested after 2 days for the reporter assay. TYR promoter activity was expressed as a percentage of the control ± SD (*P<0.01 vs. control).

Initially, we performed Western blotting to assess endogenous expression of Nrf2 and its intracellular regulator Keap1 in cultured human skin cells. We used primary cultured NHEMs, SV40T-transformed human epidermal keratinocytes (SV-HEKs) and normal human dermal fibroblasts (NHDFs). In all skin cells, expression of Nrf2 and Keap1 was detected by Western blotting (Figure 1A).

To investigate the effect of Nrf2, we created a recombinant adenovirus expressing Flag-tagged Nrf2. Adenoviral transduction did not affect the viability of NHEMs (Figure S1). After transduction of adenovirus into NHEMs, Nrf2 was highly expressed and its downstream genes such as heme oxygenase-1 (HO-1) and thioredoxin-1 (Trx-1) were markedly induced, confirming the activity of exogenously expressed Nrf2 (Figure 1B). Interestingly, overexpression of Nrf2 in NHEMs resulted in a marked decrease in pigment quantity (lane 1: 100.0%, lane 2: 81.6%±2.7, lane 3: 80.3%±11.0) (Figure 1C).

Tyrosinase is a rate-limiting enzyme for controlling the production of melanin, thus we examined the effect of Nrf2 overexpression on tyrosinase (TYR) activity. Consistent with previous data, Nrf2 overexpression led to inhibition of TYR activity compared to the control group (lane 1: 100.0%, lane 2: 55.4%±9.2, lane 3: 44.6%±1.6) (Figure 1D). Similarly, the protein levels of TYR and tyrosinase-related protein 1 (TRP1) were significantly decreased by Nrf2 overexpression. The protein level of tyrosinase-related protein 2 (TRP2, dopachrome tautomerase) was not affected (Figure 1E). To confirm whether Nrf2 overexpression inhibits transcription of the TYR gene, we co-transduced NHEMs with an Nrf2-expressing adenovirus and a TYR promoter-luciferase reporter adenovirus. As expected, Nrf2 significantly inhibited TYR promoter activity (lane 1: 99.8%±5.0, lane 2: 16.4%±0.6, lane: 17.3%±2.1) (Figure 1F). Consistent with this result, Nrf2 overexpression led to the decrease of tyrosinase mRNA level in NHEMs (Figure S2). Together, these results suggest that Nrf2 negatively regulates pigmentation of melanocytes by modulating the expression of several melanogenic enzymes.

When NHEMs were treated with Nrf2 inducers such as tert-butylhydroquinone (tBHQ), sulforaphane and curcumin, nuclear translocation of Nrf2 was observed (Figure S5). In line with the negative role for Nrf2 in pigmentation, treatment of NHEMs with curcumin resulted in downregulation of TYR (Figure S6).

**Figure 2:**
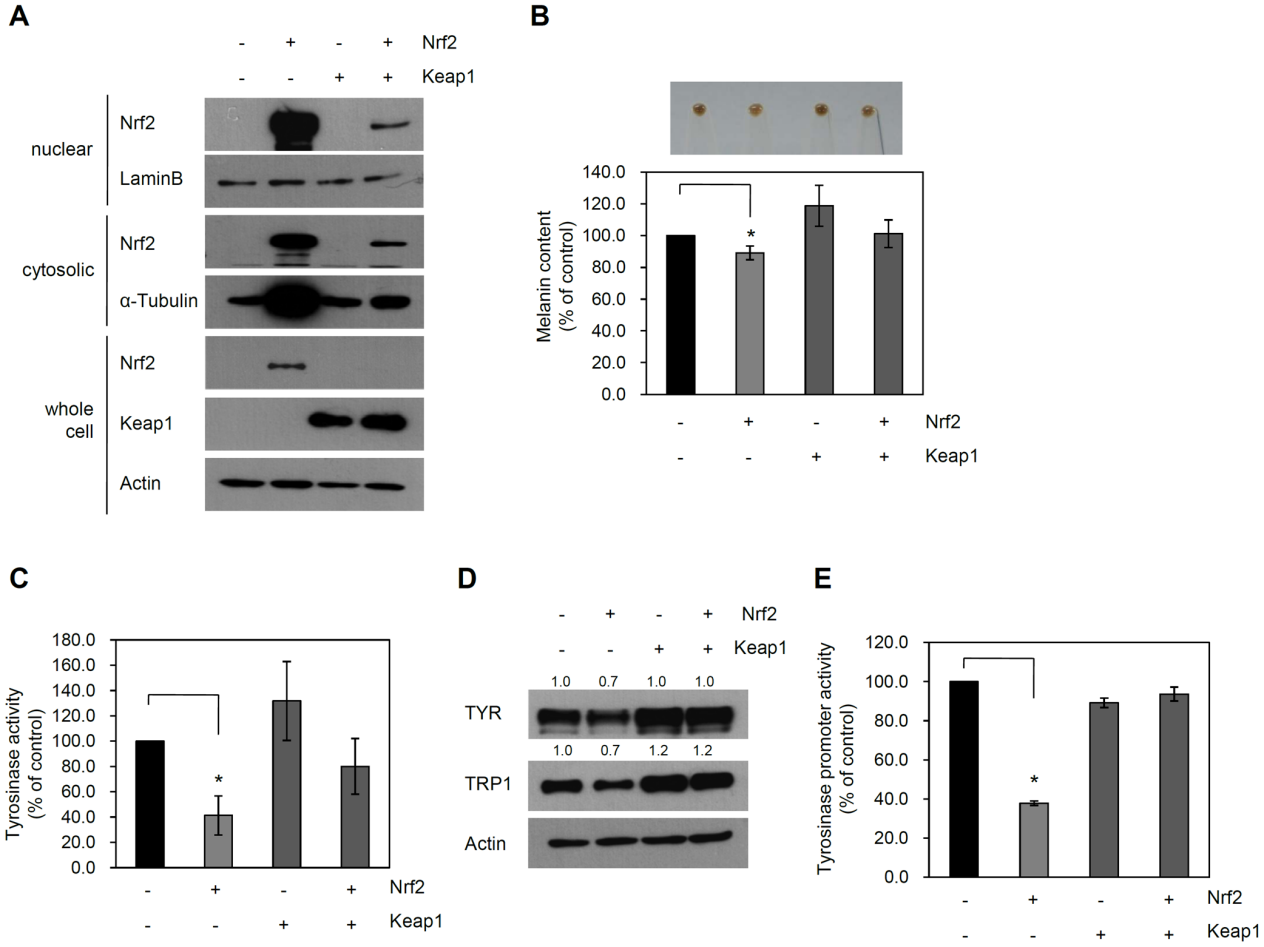
Inhibition of Nrf2-induced depigmentation by Keap1. (A) NHEMs were transduced with adenoviruses (10 MOI). Cell extracts were fractionated and Nrf2 protein was assessed by Western blotting. Lamin B was used for determination of the nuclear fraction. Whole-cell lysates were used for the detection of Keap1 and Actin. (B) NHEMs were transduced with adenoviruses (10 MOI). For detection of cell pigmentation, cells were spun down. Lower panel shows melanin content measured by spectrometer. Data are the means ± SD of triplicate measurements (*P<0.01 vs. control). (C) TYR activity was determined and expressed as a percentage of the control. Data are the means ± SD of triplicate measurements (*P<0.01 vs. control). (D) Expression of TYR and TRP1 was assessed by Western blotting. Actin was used as the loading control. (E) The TYR promoter-luciferase reporter adenovirus was co-transduced with the indicated adenoviruses. TYR promoter activity was expressed as a percentage of the control ± SD (*P<0.01 vs. control).

As Nrf2 activity is negatively regulated by Keap1, we next determined whether overexpression of Keap1 inhibited the effect of Nrf2 on melanogenesis. To this end, we co-transduced NHEMs with adenoviruses expressing Nrf2 and/or Keap1. First, we determined whether Keap1 could prevent the activation of Nrf2 in NHMCs. As Nrf2 activity is regulated by translocation to the nucleus, we assessed whether Keap1 could inhibit the nuclear translocation of Nrf2 in NHMCs. As expected, overexpression of Keap1 effectively prevented translocation of Nrf2 to the nucleus (Figure 2A). Next, we assessed the effect of Keap1 on Nrf2-induced depigmentation. As shown in Figure 2B, the Nrf2-induced decrease in melanin pigment levels was reversed by overexpression of Keap1 (lane 1: 100.0%, lane 2: 89.1%±4.3, lane 3: 118.8%±12.9, lane 4: 101.2%±8.7). In accordance with this result, Keap1 overexpression diminished Nrf2-induced downregulation of TYR activity (lane 1: 100.0%, lane 2: 42.3%±15.4, lane3: 131.7%±31.1, lane 4: 80.0±22.0) (Figure 2C). Similarly, overexpression of Keap1 reversed the effect of Nrf2 on protein levels of TYR and TRP-1 (Figure 2D). Finally, Keap1 overexpression blocked the decrease in TYR promoter activity caused by Nrf2 (lane 1: 100.0%±2.2, lane 2: 37.8%±0.8, lane3: 89.2%±5.4, lane 4: 93.6%±3.0) (Figure 2E).

We further confirmed the negative role of Nrf2 on pigmentation by knockdown experiment. To this end, we transduced NHEMs with recombinant adenovirus expressing microRNA (miR) specific for Nrf2. As shown in Figure S3, endogenous Nrf2 expression was efficiently knockdowned after transduction with adenovirus expressing miR-Nrf2. Consistent with the data obtained by Keap1 overexpression, knockdown of Nrf2 increased the protein level of tyrosinase (TYR), together with increases of TYR activity and TYR promoter activity. These results strongly support the notion that Nrf2 is a negative regulator of melanocyte pigmentation.

### Modulation of PI3K/Akt signaling by Nrf2

**Figure 3:**
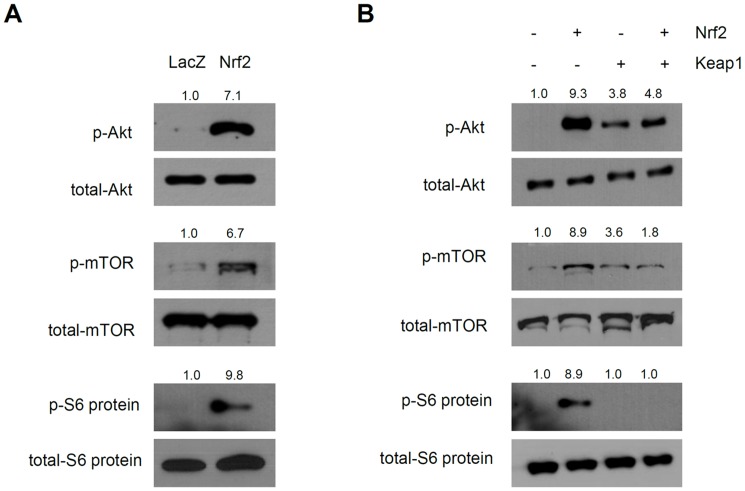
Nrf2 activates the PI3K/Akt pathway. (A) NHEMs were transduced with the indicated adenoviruses (10 MOI) and then assessed by Western blotting. Overexpression of Nrf2 induced phosphorylation of Akt, mTOR and S6 ribosomal protein. (B) Cells were transduced with the indicated adenoviruses and then assessed by Western blotting. Nrf2-induced phosphorylation was inhibited by overexpression of Keap1.

It is known that melanogenesis is regulated by the balance between a variety of signal transduction pathways, including the cyclic adenosine monophosphate/protein kinase A (cAMP/PKA), p38 mitogen-activated protein kinase (p38 MAPK), extracellular signal-regulated kinase (ERK), and phosphoinositide 3-kinase/Akt (PI3K/Akt) pathways [12]–[14], [19]. We determined the pathways related to Nrf2-induced depigmentation. The phosphorylation statuses of several signaling molecules after Nrf2 overexpression were determined by Western blotting. While we did not detect changes in the phosphorylation of CREB, p38 MAPK and ERK (data not shown), the phosphorylation of Akt was markedly increased. Moreover, the downstream molecules mTOR and S6 kinase substrate (S6 ribosomal proteins) were also activated by Nrf2 overexpression (Figure 3A). The phosphorylation of Akt, mTOR and S6 ribosomal protein in response to Nrf2 overexpression was inhibited by overexpression of Keap1 (Figure 3B), supporting the notion that Nrf2 modulates the PI3K/Akt pathway.

**Figure 4:**
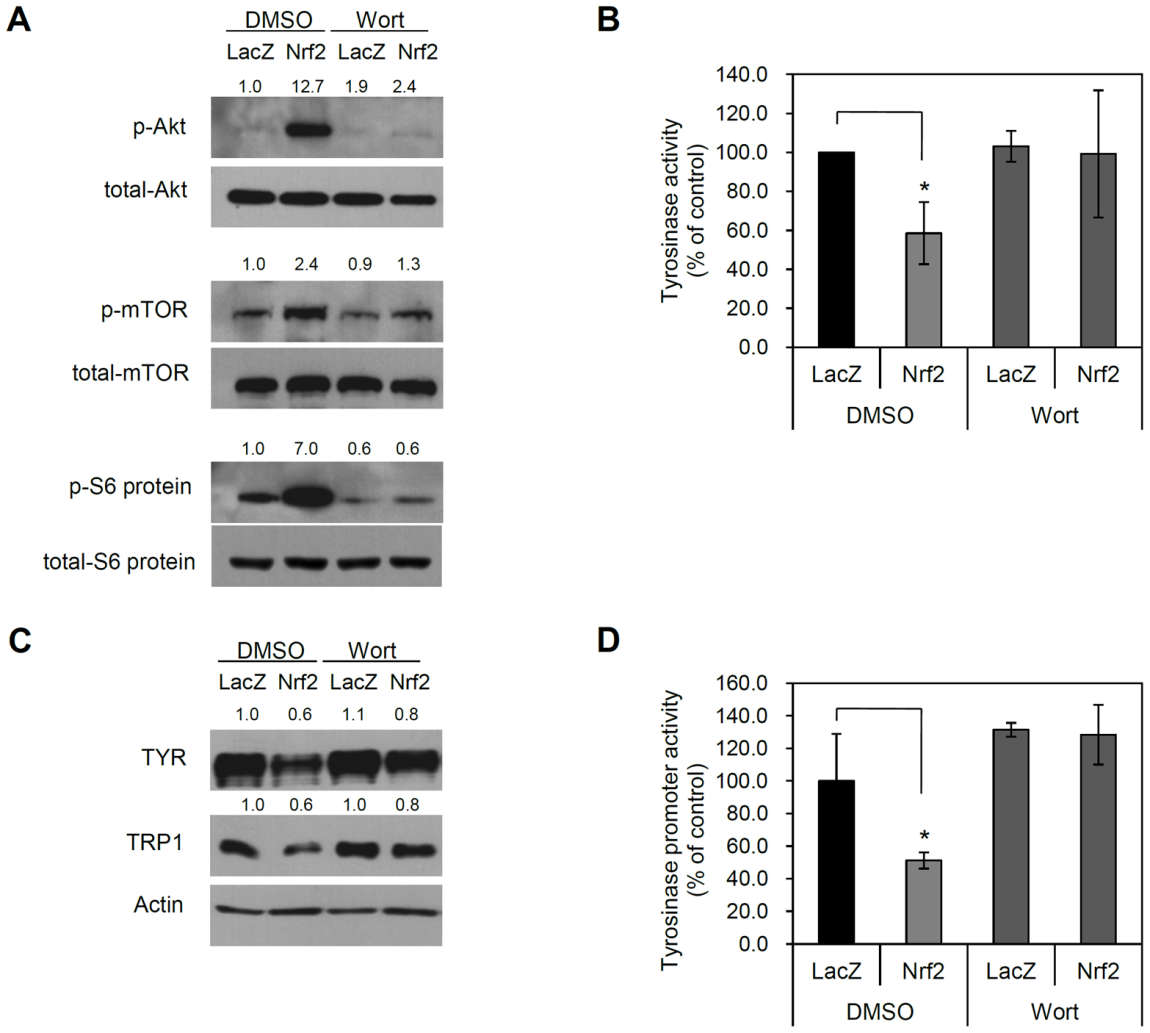
Inhibition of PI3K/AKT signaling prevents Nrf2-induced depigmentation. (A) NHEMs were transduced with the indicated adenoviruses (10 MOI) for 6 h. Cells were replenished with fresh medium containing vehicle (DMSO) or the PI3K inhibitor wortmannin (Wort, 100 nM). Cells were cultured for a 2 days, then cells were refed with fresh medium containing wortmannin again. One day after, cellular extracts were prepared and then assessed by Western blotting. Phosphorylation of Akt, mTOR and S6 ribosomal protein by Nrf2 was inhibited by PI3K inhibitor treatment. (B) Tyrosinase (TYR) activity was determined and expressed as a percentage of the control. Data are the means ± SD of triplicate measurements (*P<0.01 vs. control). Nrf2-repressed tyrosinase activity was reversed by PI3K inhibitor treatment. (C) Repression of TYR and TRP1 by Nrf2 was reversed by PI3K inhibitor treatment. (D) The TYR promoter-luciferase reporter adenovirus was co-transduced with the indicated adenoviruses. TYR promoter activity was expressed as a percentage of the control ± SD (*P<0.01 vs. control). Nrf2-repressed tyrosinase promoter activity was reversed by PI3K inhibitor treatment.

To further confirm the involvement of the PI3K/Akt signaling pathway in Nrf2-induced depigmentation of melanocytes, we treated NHEMs with the PI3K inhibitor wortmannin. As a result, PI3K inhibitor treatment effectively blocked Nrf2-induced signaling activation. Concomitantly, Nrf2-induced repression of TYR and TRP1 protein expression was reversed by PI3K inhibitor treatment (Figure 4). Finally, we performed Akt knockdown experiment using miR system to strengthen the link between Nrf2 and Akt pathway. Consistent with the data obtained by wortmannin treatment, Nrf2-induced downregulation of TYR and TRP1 was blocked by Akt knockdown (Figure S4). These results suggest that Nrf2 decreases melanogenesis by activating the PI3K/Akt pathway.

## Discussion

In this study, we demonstrated an inhibitory role for Nrf2 in melanogenesis using an adenoviral gene delivery system. Specifically, Nrf2 overexpression in NHEMs leads to significant inhibition of melanogenesis as a result of PI3K/Akt pathway activation, an inhibitory effect that is reversed by Keap1 overexpression.

It was previously demonstrated that some Nrf2 inducers, such as tert-butylhydroquinone (tBHQ), sulforaphane and curcumin, have depigmentary effects. For example, repeated dermal application of tBHQ leads to depigmentation in black guinea pigs [15]; treatment of B16 melanoma cells with sulforaphane inhibits melanogenesis and TYR expression by affecting the phosphorylation of MAP kinases [16]; and curcumin inhibits α-melanocyte stimulating hormone (α-MSH)-stimulated melanogenesis in B16 melanoma cells through the activation of MEK/ERK or PI3K/Akt [17]. We observed that Nrf2 inducers, such as tBHQ, sulforaphane and curcumin, triggered nuclear translocation of Nrf2 (Figure S5). Furthermore, we observed that Nrf2 knockdown by miR clearly prevented curcumin-induced downregulation of TYR activity (Figure S6). These results suggest that the depigmentary effects of some electrophiles may be linked to their ability to activate Nrf2.

In our study, overexpression of Nrf2 in NHEMs resulted in downregulation of TYR and TRP1 gene expression. Although Nrf2 is known to be a transcription factor that can bind to cognate DNA sequences in target genes, it is unlikely that Nrf2 directly regulates the transcription of TYR and TRP1 at the genomic level. This speculation is based on the fact that there are no known Nrf2 binding sequences in the promoters of TYR and TRP1, and that activation of Nrf2 leads to downregulation of gene expression. Thus, we hypothesized that the effect of Nrf2 may be a secondary event following gene expression and/or may be linked to the modulation of intracellular signaling. Interestingly, we found that overexpression of Nrf2 led to a robust increase in the phosphorylation of Akt. Furthermore, several signaling molecules acting downstream of Akt, such as mTOR and S6 kinase, were also activated by Nrf2. These results strongly suggest that the mechanism underlying Nrf2-induced depigmentation is modulation of intracellular signaling. This speculation is supported by previous reports that the specific PI3K inhibitors wortmannin and LY294002 stimulate melanin synthesis [18], [19]. Nonetheless, the link between Nrf2 and Akt signaling remains mysterious. It has been shown that loss of Nrf2 gene in immature dendritic cells results in increase of reactive oxygen species (ROS) [20], and intracellular ROS level certainly affects cytoplasmic signaling cascades. Thus, one possible explanation is that increased Nrf2 may affect intracellular ROS level, which in turn affects Akt signaling. Elucidation of precise signaling events underlying Nrf2-medieated depigmentation will surely be an important future study.

From dermatological and cosmetic viewpoints, the development of depigmenting and/or whitening agents is still an important issue. In this context, efforts are still focused on the development of compounds that can inhibit TYR and/or the transcription factor MITF. We suggest that the Nrf2/Keap1 pathway may be a good target for whitening agents, because the skin protective role of Nrf2 has been demonstrated. That is, non-toxic Nrf2 inducers may be cosmetically and pharmaceutically beneficial because we can expect a dual effect of Nrf2 on skin protection from UV-induced skin damage and inhibition of pigmentation.

In summary, we demonstrated a novel role for Nrf2 in the regulation of melanogenesis. Our results may contribute to a better understanding of the regulatory mechanisms of melanogenesis, and may help in the development of new targets for pigment-related skin diseases and hypopigmentation reagents.

## Materials and Methods

### Ethics Statement

This study was approved by the Institutional Review Board of Chungnam National University School of Medicine. All human skin samples were obtained under the written informed consent of donors.

### Cell culture

Foreskin specimens were briefly sterilized in 70% ethanol, minced, and then treated with dispase for overnight at 4°C. The epidermis was separated and placed in a solution containing 0.05% trypsin and 0.02% ethylenediaminetetraacetic acid (EDTA) (Gibco BRL, Rockville, MD) for 30 min at 37°C. After vigorous pipetting, cells were pelleted and resuspended in growth medium, which is composed of Medium 254 and human melanocyte growth supplement (Cascade Biologics, Portland, OR). During the primary culture of human melanocytes, 200 µg/ml of G418 (geneticin sulfate, Duchefa, Haarlem, The Netherlands) was added to the growth medium to suppress the proliferation of fibroblasts [21]. For determining the effect of electrophiles, NHEMs were treated with tert-butylhydroquinone (tBHQ), sulforaphane and curcumin. All chemicals were obtained from Sigma-Aldrich (St. Louis, MO) and dissolved in dimethyl sulfoxide (DMSO).

### Production of recombinant adenovirus

Total RNA was isolated from cultured skin epithelial cells using Easy-blue RNA extraction kit (Intron, Daejeon, Korea). Two μg of total RNA was reverse transcribed with moloney-murine leukaemia virus (M-MLV) reverse transcriptase (ELPIS Biotech, Daejeon, Korea). Aliquot of RT mixture was subjected to PCR cycles with primer set for Nrf2 (5’-GGAAAGACGGGTACCATGATGGACTTGGAGCTG-3’, 5’-CCGTAACACCTCGAGCTAGTTTTTCTTAACATC-3’), and Keap1 (5’-AAAGGATCCATGCAGCCAGATCCCAGGCC-3’, 5’-CCCTCGAGTCAACAGGTACAGTTCTGCTGG-3’). The Nrf2 and Keap1 cDNAs were subcloned into pENTR/CMV-Flag vector that has attL sites for site-specific recombination with a Gateway destination vector (Invitrogen, Carlsbad, CA). The replication-incompetent adenoviruses were created using Virapower adenovirus expression system (Invitrogen) according to the method previously described [22]. Briefly, site-specific recombination between entry vector and adenoviral destination vector was achieved by LR clonase (Invitirogen). The resulting adenoviral expression vector was then transfected into 293A cells using Lipofectamine 2000 (Invitrogen). Cells were grown until 80% cytopathic effect (CPE) was seen, then harvested for preparation of recombinant adenovirus.

For miR specific for Nrf2 and Akt1, target sequences were designed using Invitrogen's RNAi Designer (http://rnaidesigner.lifetechnologies.com/rnaiexpress). The double-stranded DNA oligonucleotides were synthesized and cloned into the parental vector pcDNA6.2-GW/EmGFP-miR (Invitrogen). The expression cassette for miR was moved into pENT/CMV vector, and then adenovirus was made. The miR sequences were as follows: Nrf2, top strand 5'- TGCTGTAGATCAGAAACATCAATGGGGTTTTGGCCACTGACTGACCCCATTGATTTCTGATCTA-3' and bottom strand 5'- CCTGTAGATCAGAAATCAATGGGGTCAGTCAGTGGCCAAAACCCCATTGATGTTTCTGATCTAC-3'; Akt1, top strand 5'- TGCTGTGGAAGGTGCGTTCGATGACAGTTTTGGCCACTGACTGACTGTCATCGCGCACCTTCCA-3' and bottom strand 5'- CCTGTGGAAGGTGCGCGATGACAGTCAGTCAGTGGCCAAAACTGTCATCGAACGCACCTTCCAC-3'.

### Western blot analysis

Cells were harvested by centrifugation and then lysed in protein extraction solution (Intron). After vigorous pipetting, extracts were centrifuged for 15 min at 15,000 rpm. Total protein was measured using a BCA protein assay kit (Thermo Scientific, Rockford, IL). Samples (20–30 µg protein per lane) were run on SDS-polyacrylamide gels, transferred onto nitrocellulose membranes and incubated with appropriate antibodies for overnight at 4°C with gentle agitation. Blots were then incubated with peroxidase-conjugated secondary antibodies for 30 minutes at room temperature, and visualized by enhanced chemiluminescence (Intron). The following primary antibodies were used in this study: Nrf2, Keap1, tyrosinase, TRP1, TRP2, actin and laminB (Santa Cruz Biotechnologies, Santa Cruz, CA), phospho Akt, total Akt, phospho mTOR, total mTOR and phospho S6 ribosomal proteins (Cell Signaling Technology, Danvers, MA).

### Subcellular fractionation

Nuclear extracts from the cells were prepared using NE-PER Nuclear and Cytoplasmic Extraction Reagents (Thermo Scientific), according to the recommended protocol. To confirm the purity of subcellular fractionation, the extracts were Western blotted and probed with nuclear specific anti-laminB antibody.

### Melanin content and Tyrosinase activity

For determination of pellet pigmentation, cells were collected and pelleted by centrifugation. Melanin pigment was dissolved in 1 N NaOH at 100°C for 30 min, and quantified by measuring optical density at 405 nm. Tyrosinase activity was determined as described previously with slight modification [23]. Briefly, cells were lysed in Pro-Prep protein extraction solution (Intron), then lysate was clarified by centrifugation. After quantification, 250 µg of total protein in 100 µl of lysis buffer was transferred into the 96-well plate, and 100 µl of 1 mM L-DOPA was added. After incubation for 30 min at 37°C, absorbance was measured at 405 nm. The tyrosinase activity was expressed as a percentage to control. All experiments were done at least three times.

### Luciferase assay

Cells were grown at 50% confluency in 6-well culture plate, then co-transduced with adenovirus harboring TYR promoter reporter cassette and adenoviruses expressing Flag-tagged Nrf2 and/or Flag-tagged Keap1. After incubation for 6 h, cells were replenished with fresh medium. Cells were further incubated for 2 days. Luciferase activities were determined using Luciferase assay system (Promega, Madison, WI), according to the recommended protocol.

### Statistical analysis

Data were evaluated statistically using one-way ANOVA using SPSS software (ver 22.0). Statistical significance was set at p<0.01.

## Supporting Information

Figure S1 **Cell viability after adenoviral transduction.** NHEMs were transduced with an adenovirus expressing Nrf2 or LacZ (control) at the indicated multiplicity of infection (MOI) for 6 h. Cells were replenished and then cultured for a further 3 days. Cell viability was determined by MTT assay.
(PDF)

Figure S2 **NHEMs were transduced with an adenovirus expressing Nrf2 or LacZ (control) at the indicated multiplicity of infection (MOI) for 6 h.** Cells were replenished and then cultured for a further 3 days. The mRNA levels were evaluated by RT-PCR. CYP (cyclophilin) was used as loading control.
(PDF)

Figure S3 **NHEMs were transduced with adenovirus expressing microRNA-Nrf2 (miR-Nrf2) or scrambled control (miR-scr) at the 10 multiplicity of infection (MOIs) for 6 h.** Cells were replenished and then cultured for a further 3 days. Effect of Nrf2 Knockdown on melanogenesis is determined by (A) Western blotting, (B) TYR activity and (C) TYR promoter activity. (*P<0.01 vs. control).
(PDF)

Figure S4 **NHEMs were co-transduced with indicated adenoviruses at the 10 MOIs for 6 h.** Cells were replenished and then cultured for a further 3 days. Expression of Nrf2 and pigmentation-related genes was determined by Western blot. Kockdown of Akt by miR significantly prevented Nrf2-induced downregulation of TYR and TRP1.
(PDF)

Figure S5 **NHEMs were treated with Nrf2 inducers such as tert-butylhydroquinone (tBHQ), sulforaphane and curcumin, for the indicated time points.** Total cell pellets were separated into cytosolic and nuclear fraction. Nrf2 translocation was determined by Western blot. To confirm the purity of subcellular fractionation, the extracts were probed with cytosol specific anti-α-tubulin and nucleus specific anti-laminB antibody. All experiments were performed three times. M: marker.
(PDF)

Figure S6 (A) NHEMs were treated with curcumin at the indicated concentrations for 3 days. Expression of Nrf2, tyrosinase (TYR), and phospho-Akt was determined by Western blot. (B) NHEMs were transduced with adenovirus expressing miR-Nrf2 or miR-scr. Cells were replenished and treated with curcumin (12.5 µM) for 3 days. TYR activity was determined and expressed as a percentage of the control. Data are the means ± SD of triplicate measurements (*P<0.01 vs. control).
(PDF)
