# Supplementary figures and images for: Nrf2 Negatively Regulates Melanogenesis by Modulating PI3K/Akt Signaling
Source: PLoS One. 2014 Apr 24;9(4):e96035. doi: 10.1371/journal.pone.0096035 (PMC3999113; doi:10.1371/journal.pone.0096035)

Figure S1

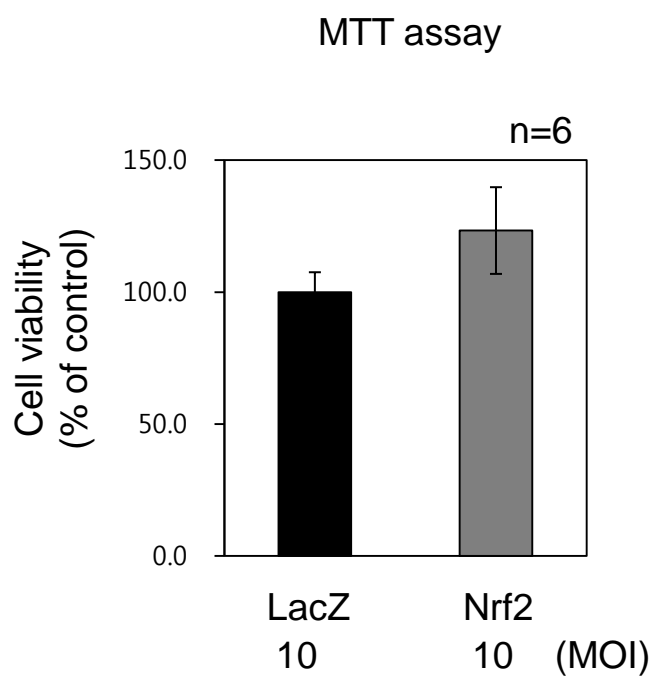

Supplement: Figure S1 — Cell viability after adenoviral transduction. NHEMs were transduced with an adenovirus expressing Nrf2 or LacZ (control) at the indicated multiplicity of infection (MOI) for 6 h. Cells were replenished and then cultured for a further 3 days. Cell viability was determined by MTT assay. (PDF) [file pone.0096035.s001.pdf]

Figure S2

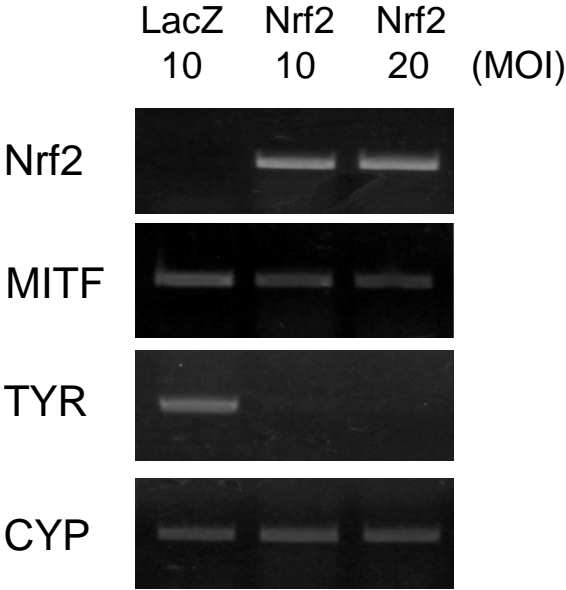

Supplement: Figure S2 — NHEMs were transduced with an adenovirus expressing Nrf2 or LacZ (control) at the indicated multiplicity of infection (MOI) for 6 h. Cells were replenished and then cultured for a further 3 days. The mRNA levels were evaluated by RT-PCR. CYP (cyclophilin) was used as loading control. (PDF) [file pone.0096035.s002.pdf]

Figure S3

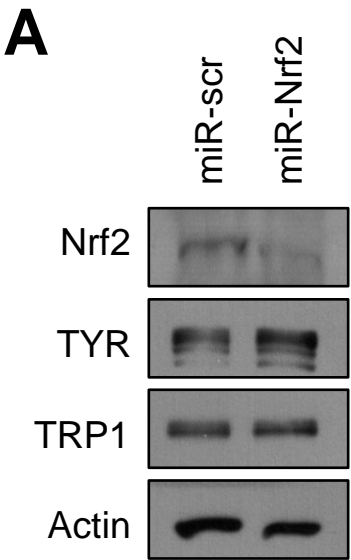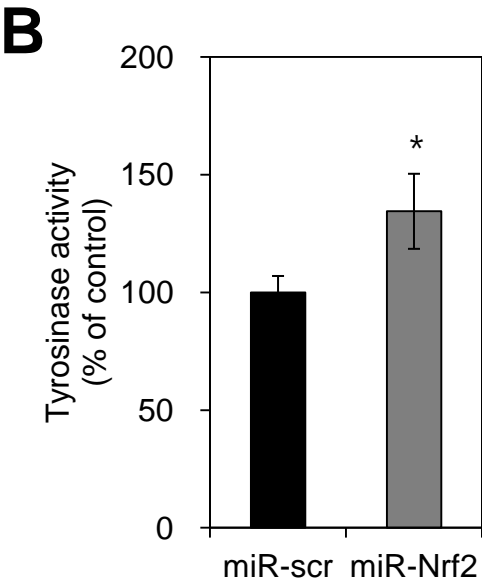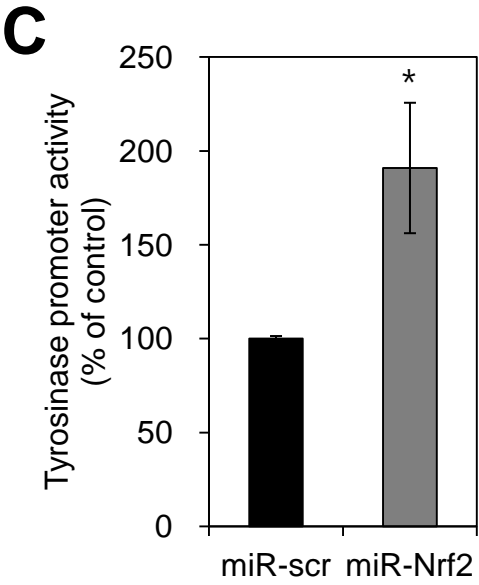

Supplement: Figure S3 — NHEMs were transduced with adenovirus expressing microRNA-Nrf2 (miR-Nrf2) or scrambled control (miR-scr) at the 10 multiplicity of infection (MOIs) for 6 h. Cells were replenished and then cultured for a further 3 days. Effect of Nrf2 Knockdown on melanogenesis is determined by (A) Western blotting, (B) TYR activity and (C) TYR promoter activity. (*P<0.01 vs. control). (PDF) [file pone.0096035.s003.pdf]

Figure S4

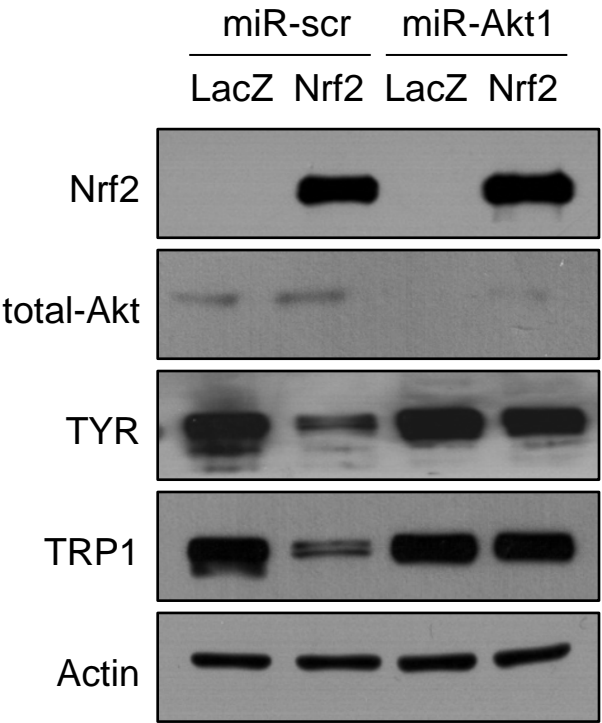

Supplement: Figure S4 — NHEMs were co-transduced with indicated adenoviruses at the 10 MOIs for 6 h. Cells were replenished and then cultured for a further 3 days. Expression of Nrf2 and pigmentation-related genes was determined by Western blot. Kockdown of Akt by miR significantly prevented Nrf2-induced downregulation of TYR and TRP1. (PDF) [file pone.0096035.s004.pdf]

Figure S5

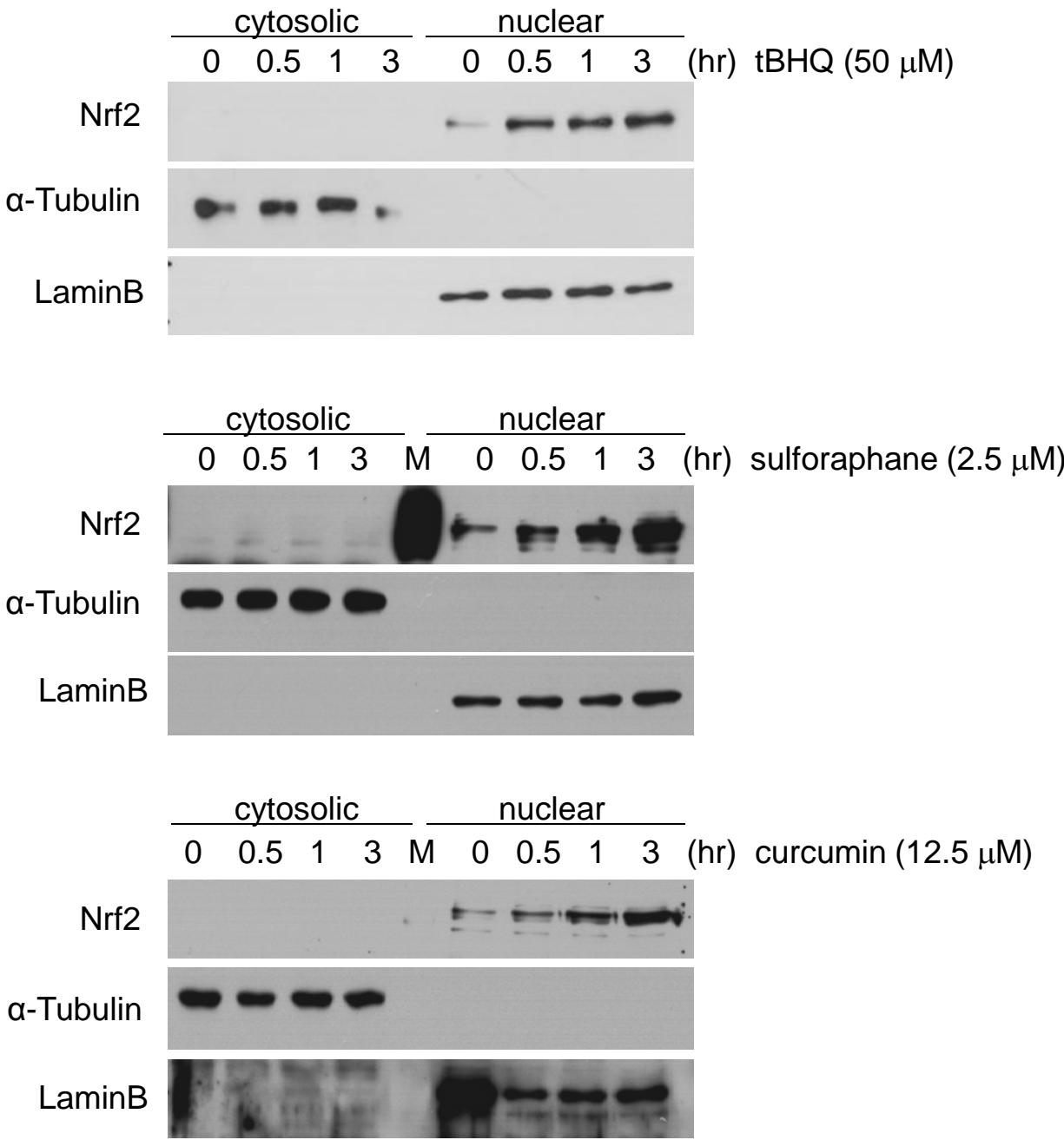

Supplement: Figure S5 — NHEMs were treated with Nrf2 inducers such as tert-butylhydroquinone (tBHQ), sulforaphane and curcumin, for the indicated time points. Total cell pellets were separated into cytosolic and nuclear fraction. Nrf2 translocation was determined by Western blot. To confirm the purity of subcellular fractionation, the extracts were probed with cytosol specific anti-α-tubulin and nucleus specific anti-laminB antibody. All experiments were performed three times. M: marker. (PDF) [file pone.0096035.s005.pdf]

Figure S6

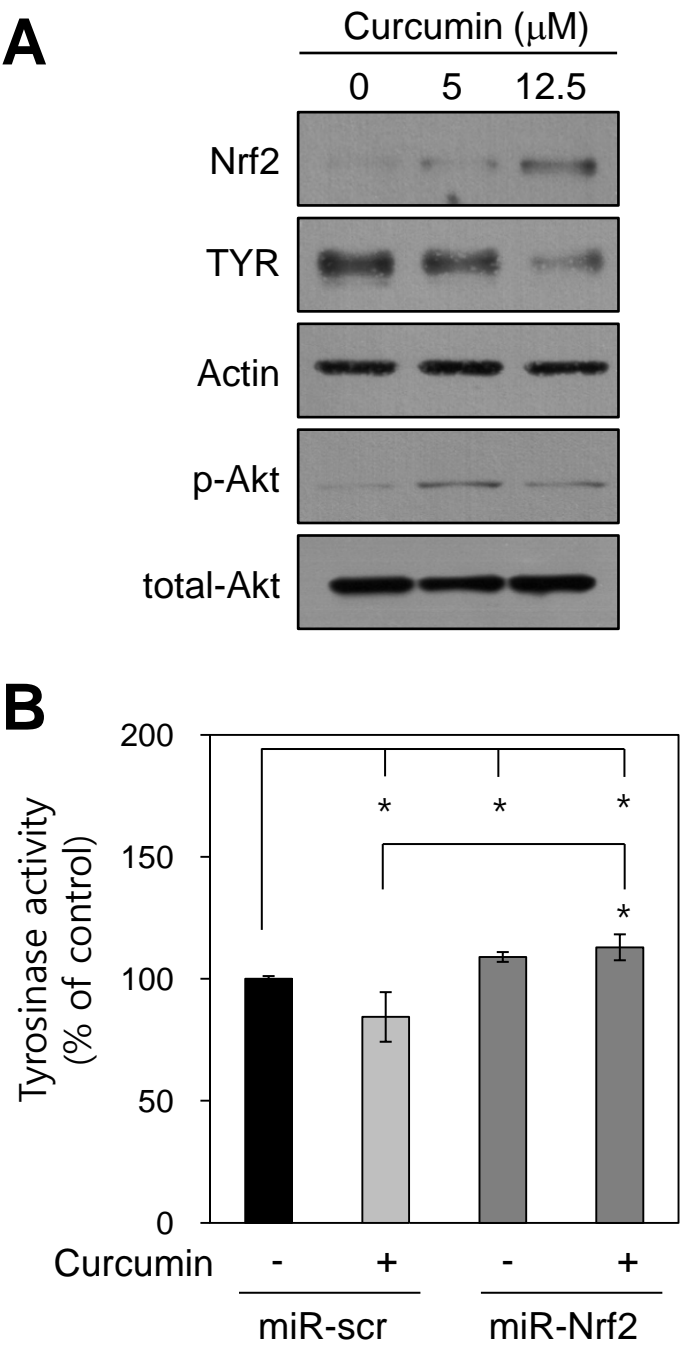

Supplement: Figure S6 — (A) NHEMs were treated with curcumin at the indicated concentrations for 3 days. Expression of Nrf2, tyrosinase (TYR), and phospho-Akt was determined by Western blot. (B) NHEMs were transduced with adenovirus expressing miR-Nrf2 or miR-scr. Cells were replenished and treated with curcumin (12.5 µM) for 3 days. TYR activity was determined and expressed as a percentage of the control. Data are the means ± SD of triplicate measurements (*P<0.01 vs. control). (PDF) [file pone.0096035.s006.pdf]
